# Spatial Genetic Structure of a Symbiotic Beetle-Fungal System: Toward Multi-Taxa Integrated Landscape Genetics

**DOI:** 10.1371/journal.pone.0025359

**Published:** 2011-10-04

**Authors:** Patrick M. A. James, Dave W. Coltman, Brent W. Murray, Richard C. Hamelin, Felix A. H. Sperling

**Affiliations:** 1 Department of Biological Sciences, University of Alberta, Edmonton, Alberta, Canada; 2 Natural Resources and Environmental Studies Institute, University of Northern British Columbia, Prince George, British Columbia, Canada; 3 Department of Forest Sciences, University of British Columbia, Vancouver, British Columbia, Canada; 4 Natural Resources Canada, Québec, Canada; Montreal Botanical Garden, Canada

## Abstract

Spatial patterns of genetic variation in interacting species can identify shared features that are important to gene flow and can elucidate co-evolutionary relationships. We assessed concordance in spatial genetic variation between the mountain pine beetle (*Dendroctonus ponderosae*) and one of its fungal symbionts, *Grosmanniaclavigera*, in western Canada using neutral genetic markers. We examined how spatial heterogeneity affects genetic variation within beetles and fungi and developed a novel integrated landscape genetics approach to assess reciprocal genetic influences between species using constrained ordination. We also compared landscape genetic models built using Euclidean distances based on allele frequencies to traditional pair-wise *Fst*. Both beetles and fungi exhibited moderate levels of genetic structure over the total study area, low levels of structure in the south, and more pronounced fungal structure in the north. Beetle genetic variation was associated with geographic location while that of the fungus was not. Pinevolume and climate explained beetle genetic variation in the northern region of recent outbreak expansion. Reciprocal genetic relationships were only detectedin the south where there has been alonger history of beetle infestations. The Euclidean distance and *Fst*-based analyses resulted in similar models in the north and over the entire study area, but differences between methods in the south suggest that genetic distances measures should be selected based on ecological and evolutionary contexts. The integrated landscape genetics framework we present is powerful, general, and can be applied to other systems to quantify the biotic and abiotic determinants of spatial genetic variation within and among taxa.

## Introduction

The current mountain pine beetle (MPB; *Dendroctonusponderasae*) outbreak in western Canada is unprecedented in terms of extent and severity and has had significant ecological and economic consequences [1], [2]. Bark beetle outbreaks are the product of complex interactions among an endemic bark beetle, symbiotic pathogenic fungi (e.g., *Ophiostoma spp.*), host trees (*Pinus spp.*), landscape features, and climate [3], [4]. Host tree mortality is the result of the combined effects of beetle damage and fungi-induced water stress [5], [6]. Examination of the spatial apportionment of genetic variance in these two species and how their respective genomes are correlatedcan be useful in predictingoutbreak risk and in understanding coevolution between bark beetles and their associated fungi.

The mountain pine beetlehas a symbiotic relationship with several fungi in the Ophiostomataceaefamily [7]. These fungi provide benefits to the beetle including larvalnutrition, protection from tree defenses, and stressing attacked trees to facilitate beetle mass attack [5], [8], [9]. Likewise, the MPB provide the fungus with a dispersal mechanism and access to a tree's conductive tissues [10], [11]. Dispersal of fungal spores by the MPB can occur actively through transport in highly specialized mycangia, or passively through incidental transport on the beetle exoskeleton [10]. Previous work has investigated the phylogenetic history of this beetle-fungal symbiosis [12] but no studies have yet examined or compared the contemporary population genetic structure in these two species. Otherstudies have compared the contemporary genetic structure of interacting species including termites and symbiotic fungi [13], ants and their cultivated fungi [14], and other host-parasitoid interactions [15]. However, few of these studies have compared contemporary genetic variation of symbionts in a spatially explicit context, although the importance of spatial heterogenetiy to species interactions and coevolution is well accepted [15], [16].

**Figure 1:**
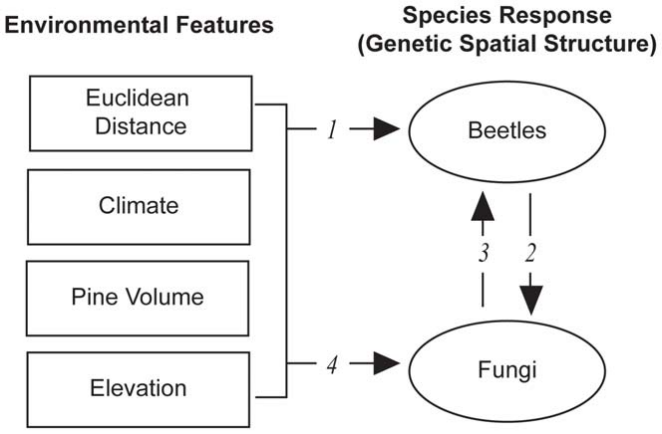
Conceptual depiction of integrated landscape genetics framework in which multiple paths may connect environmental features and the spatial genetic structure of the mountain pine beetle and *G. clavigera*. Our objective was to characterize and quantify these numbered paths using constrained ordination and model selection. Paths for which we found support are summarized in Table 5.

In this study we compare and contrast spatial genetic variation in the mountain pine beetle and its primary fungal symbiont *G. clavigera*. We assess the extent and pattern of genetic structure in each species, whether these patternsin genetic variability were associated with geographic location, andwhether these patterns were similar between species. Then, using a landscape genetics approach [17], [18], we investigate whether landscape features help explain observed genetic variation in each species. Finally, we extend the landscape genetics framework to include biotic variables and use beetle and fungal genotypes in combination with landscape features to model genetic variation of both species. This novel ‘integrated landscape genetics’ framework allows us to test the hypothesis that genetic variation within one symbiont can be used to predict the genetic structure of the other (Figure 1). Here, the reciprocal interactions between taxa can be thought of as another type of ‘landscape’ that canfacilitate or constrain gene flow and hence influence spatial genetic variation similar to an “extended phenotype” [19]. Our goal was to partition genetic variance in both species that is explained by spatial, environmental, and geneticfactors and to test the strength of the different pathways among environmental and genetic variables using constrained ordination (Figure 1).

Genetic variation of interacting species may be correlated for several reasons: (1) genomes may interact directly through the selective advantage of particular combinations of alleles (i.e., coevolution), (2) genomes may appear correlated because species share similar life histories and/or movement patterns (e.g., symbioses), or (3) genomes may appear correlateddue to indirect factors such as shared responses to environmental heterogeneity (e.g., spatial dependence). Because microsatellite markers aregenerally not correlated with adaptive variation [20], we focus on the second and third possibilities. Here, the genetic structure of the interacting symbionts acts as a surrogate variable for unmeasured environmental or demographic processes. Indentifying relationships between species movement and spatial heterogeneity through the integration of environmental and genomic variation is of fundamental importance to understanding and predicting spatial population dynamics in systems with tightly coupled and interacting species or communities [21].

## Methods

### Study Area and Sampling

**Figure 2:**
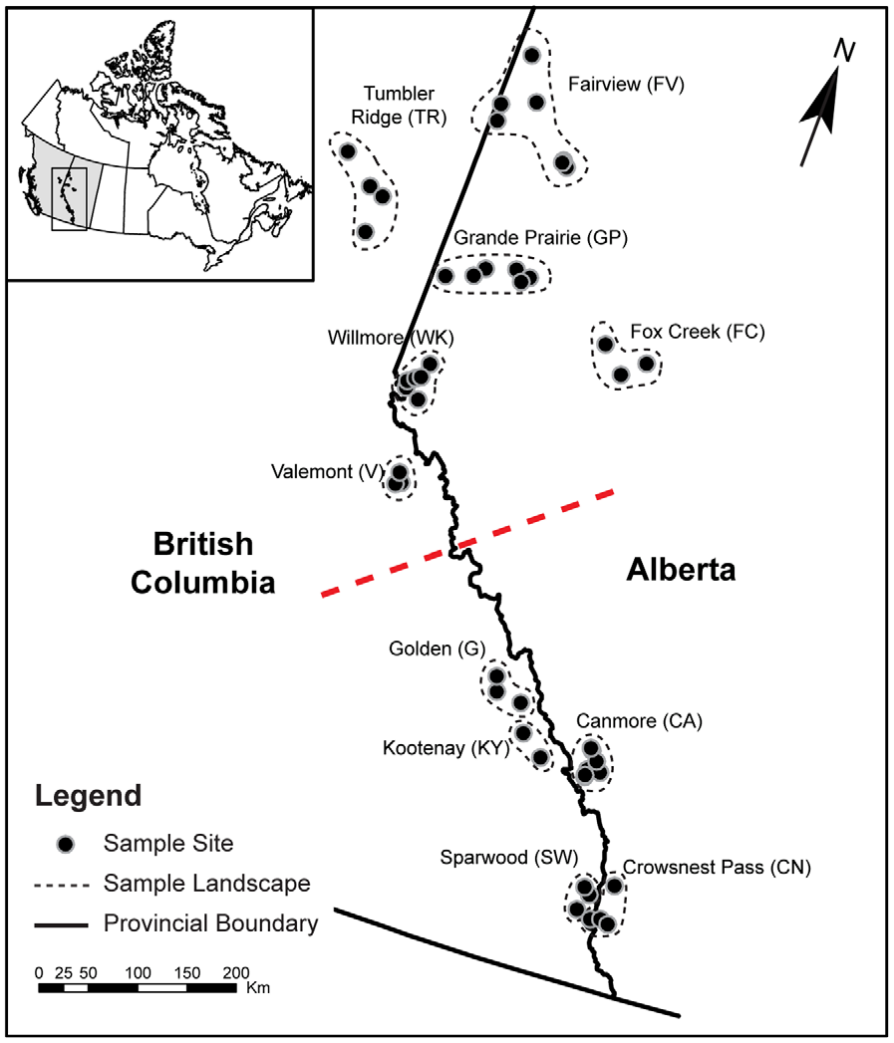
Location and regional context of sample landscapes comprised of multiple sites from which beetle and fungal genotypes were obtained. Dashed line separates the northern and southern regions. Details of each sample landscape are summarized in the Table S1.

Beetles and fungi were collected in Alberta and British Columbia, Canada in two sample periods (Figure 2, Table S1) from visibly attacked mature lodgepole pine (*Pinuscontorta*) and hybrid jack (*Pinusbanksiana*)-lodgepole pine trees. Permits were obtained when required to cover both the collection and transport of beetle and fungal materials. Parks Canada provided permits for the mountain parks Kootenay and Yoho. Alberta Tourism, Parks, and Recreation provided permits for collection in the Wilmore Wilderness, Cypress Hills and around Canmore (Kananaskis). We also received permission from Tembec to collect in their forest around Sparwood.

There were three levels to the sampling design: (1) landscapes, (2) sites within landscapes, and (3) individual trees within sites (Figure 2; Table S1). Not all locations were sampled in both periods. Landscapes were selected to represent a range of ecoregions [22] at the leading edge of the MPB outbreak, with up to five sites sampled in each landscape at a minimum of 5 km apart (Table S1). The study area is approximately 158 000 km^2^, and extends from 49.6° to 56.9°north latitude and 114.4° to 121.9° west longitude. Beetle larvae and adults were sampled from galleries within 5″ phloem disks extracted from individual trees. Full details onspecimen collection are found in Roe *et al*. [23] and details on sampling locations are reported in the Table S1. In addition to examining the entire study area, we examined twosub-regions, a northern and asouthern, which reflect the progression of the recent outbreak [2], [24].

In total, 2943 individual beetles and 174single spore fungal isolates were collected from 44sites within 16 landscapes. Fungal data were corrected for clonality by removing individuals with identical microsatellite profiles at all loci which resulted in 156 unique isolates. Analysis was performed on allele frequenciesor matrices of pair-wise *Fst* values summarized to the level of the landscape to ensure adequate sample sizes for both species. Werestricted our analysis to landscapes for which both fungal and beetle samples were available. Fungi were sampled from galleries and adjacent wood tissue [23]. We assessed whether there were differences in the level of genetic structure of fungi isolated from both sources using AMOVA (Smouse et al 2001). We pooled samples from different sources and/or years if fungal source was not was not a statistically significant predictor of molecular variance. Also, some landscapes were sampled for beetle and fungi in both sample periods (GP, WK, CA, CN; Table S1). We assessed the significance of year of sampling in these locations using AMOVA to rule out the possibility that observed spatial patterns were due to temporal variation. All AMOVA were carried out using Arlequinv3.5.1.2 [25].

### Molecular Markers

We used 13 microsatellite loci to identify genetic structure in the mountain pine beetle [26] and 7 microsatellite locifor *G. clavigera* [27]. All markers were in Hardy-Weinbergand linkage equilibrium. Allele frequencies were calculated for beetles and fungi within each landscape and geo-referenced to the centroid of the minimum convex polygon surrounding the sites that comprise each landscape. Frequencies were standardized by the lowest value and natural log-transformedwhile retaining zero values. Log-transformation is necessary to account for statistical non-independence among alleles within each locus [28].

### Genetic Structure

Genetic structure for each species was assessed using a global estimate of *Fst* and the landscape as the sample units [29]. We then used AMOVA [30] to partition fungal and beetle genetic variation among the sample landscapes and the northern and southern regions of the study area (Figure 2). We tested for isolation by distance through plots of *Fst* against geographic distance (log-transformed) for the entire study area and within the northern and southern regions.

Variation in allele frequencies among landscapes for each species was summarized and visualized using principal components analysis (PCA) calculated using the *rda* function in the veganpackage in R [31]. To assess concordance between beetle and fungal spatial genetic variation we used a Procrustesrotationtest, also known as co-inertia analysis, [32], which is similar to the Mantel test in that it assessessimilarity between multivariate data tables. The difference between this approach and the Mantel test is that the analysis is performed on the raw data or their ordination solutions, rather than derived distance matrices, and is as a result more powerful [32]. Partial analyses are also possible where the effects of Euclidean distanceare ‘removed’ prior to analysis through partial linear regression. We assessed congruence between beetle and fungal ordinations of (1) raw allele frequency data; and (2) frequency data that were detrended with respect to Euclidean connectivity among sites. Tests were performed within the northern and southern regions independently, as well as for the entire study area using the *protest* function in the vegan package in R [31].

PCA calculates distances among sites in multi-dimensional space based on allele frequencies but does not make many of the evolutionary assumptions of more traditional distance measures such as *Fst* [33]. Given that the mountain pine beetle in our study were in outbreak phase, and their recent and rapid spread into northern British Columbia and Alberta [2], [24], many of these assumptions are likely to be violated [33]. However, to assess its performance relative to*Fst* [34], we compared the PCA ordination to a principal coordinates analysis (PCoA) of pair-wise *Fst* values. PCoA summarizes and plots differences among sites based on an input distance matrix [35]. Concordance between the ordination solutions of the different methods (PCA vs. PCoA with *Fst*) was assessed using a Procrustestest, as described above. Pair-wise *Fst* values (Nei 1973) were calculated using the *pairwise. fst* function in the adegenet package in R [36]. Principle coordinates (PCoA) were calculated using the *capscale* function in the vegan package in R [31].

### Landscape Genetics

After characterizing genetic structure in both species, we investigated whether landscape features could explain further marginal variation in allele frequencies among landscapes using redundancy analysis (RDA), a form of constrained ordination. RDA is the canonical extension to PCA in whichthe principal components produced are constrained to be linear combinations of a set of predictor (environmental) variables [37]. The objective of this analysis was to identify the best ordination modelsthat describe genetic similarities among landscapesin the different regionsto better understand how spatial heterogeneity affectsgene flow in the mountain pine beetle and *G. clavigera*.

Within RDA models we used log-transformed allele frequencies as the response matrixand several measures of spatial connectivity as predictors. Predictor variablesincluded connectivity among locations based on geographic (Euclidean) distance, elevation, climatic suitability [38], and pine volume [39], [40]. All environmental predictor variables were standardized to a zero mean and unit variance [37]and detrendedsuch that environmental connectivity was independent of Euclidean connectivity. The spatial genetic structure of each symbiont, represented by the first and second principal component scores (PC1 and PC2) from the respective original PCAs, wasalso included to assess whether symbiontscan help to explain each other's genetic structure. The PC variables are location-based, as opposed to connectivity based, and were not detrended. The best model comprised of significant predictors was selected using forward selection with permutation and an inclusion threshold of α = 0.05 using the *ordistep* function of the vegan package in R [31]. Forward selection refers to a process to build parsimonious statistical models in which successive predictors are added to a model and assessed as to whether they significantly improve model fit [35]. When more than one predictor was found to be significant in a given model, we used variance partitioning [35] to identify the unique and shared contributions of each significant predictor to variance explained using the *varpart* function in vegan.

Similar to the comparison between PCA and an *Fst*-based PCoAdescribed above, we compared models constructed using: (1) RDA in which Euclidean distances are the response variable, and (2) distance-based redundancy analysis (dbRDA; [41]) in which pair-wise *Fst* values are used as the response matrix. In the case of dbRDA, the spatial genetic structure of each species was represented among the set of predictors as the first and second PCoA axes. Models were similarly selected using forward selection.

### Spatial Connectivity

A challenge to using constrained ordination in landscape genetics studies is to convert spatial environmental data into location-specific information rather than measures of dissimilarity as are often employed in Mantel testing frameworks [42]. We measuredthe connectivity of each site to the rest of the network based on resistance surfaces that represent hypotheses of the influence of different landcover types to beetle movement. Here, connectivity within the network of sample locations for each site *i* (*S_i_*) was calculated using a general ecological connectivity metric often used in meta-population studies [43].

Connectivity of a site was represented as the average connectivity of that point to all othersites in the network for a given resistance surface. Connectivity for each site for each of the four resistance surfaces (Table 1) was calculated as:
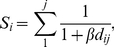
(1)where *S_i_* is the connectivity of patch *i* to the rest of the points in the network (*j*), *d_ij_* is the effective distance calculated from the least-cost path between points *i* and *j* based on the resistance surface, and *β* is the estimated mean beetle dispersal distance that parameterizes a kernel that weights a connection between a given pair of locations.

**Table 1:** Summary of predictor variables used in constrained ordinations.

Data	Source	Original Resolution	Connectivity Based?	Why chosen	Ref.
Elevation	ASTER DEM^1^	30 m.	Yes	Hypothesized beetle dispersal limitation at high elevations.	[58]
Climatic Suitability Index (CSI)	CFS^2^	1000 m.	Yes	Demonstrated climatic limitations to successful reproduction.	[38], [53]
Pine Volume	CFS^3^	1000 m.	Yes	Beetles preferentially attack large diameter trees and high-volume stands.	[24], [39]
Geographic Dist.	Calculated	1000 m.	Yes	Null hypothesis - Isolation By Distance.	[59]
Beetle PC1& PC2^4^	Initial PCA	NA	No	Hypothesis based on known symbiosis.	[7]
Fungus PC1& PC2^4^	Initial PCA	NA	No	Hypothesis based on known symbiosis	[7]

1 NASA DEM data were accessed through https://wist.echo.nasa.gov/api/.

2 Climate suitability data were obtained through the Canadian Forest Service (CFS).

3 Yemshanov, D, McKenney, D, Pedlar, J. *in review*. Mapping forest composition from the Canadian National Forest Inventory and satellite landcover classification maps. Environmental Monitoring and Assessment.

4 The first and second axes of a principal coordinates analysis using *Fst* (PCoA) were used in distance based redundancy analysis (dbRDA).

The mountain pine beetle is capable of long-distance dispersal [44]. However, the frequency of long-distance dispersal is uncertain and difficult to measure [39], [44]. Long-distance dispersal is thought to be the result of convective air movements that coincide with beetle flight periods that allow passive long-distance movement in the atmospheric boundary layer [44]. For this study, *β* was set to 40 km to accommodate recently observed dispersal events of greater than 100 km that brought beetles over the Rocky Mountains into Alberta [24], [44].

### Resistance Surfaces

Resistance surfaces (Table 1) were used to calculate least-cost effective distances between sample locations and connectivity among them. Least-cost effective distances measure the degree of resistance of a landscape with respect to an environmental variable along a least-cost path [45]. We calculated pair-wise effective distances between sites using the ‘spatial graphs’ package in SELES [46] and estimated consequent connectivity for each location and resistance surface using equation 1. High values for elevation were hypothesized to restrict beetle movement while high pine volume was hypothesized to facilitate movement and connectivity. Climatic suitability was modelled using aclimatic suitability index [38] that integrates number of degree days above 5.5°C, minimum winter temperatures, maximum August temperatures, precipitation, and aridity. Greater climatic suitability was also hypothesized to facilitate movement. Resistance surfaces were composed of raw continuous values that represented the landscape feature without reclassification, thus maintaining the highest thematic resolution possible (Cushman and Landguth 2010). Spatial resolution of all resistance surfaces was 1 km.

## Results

### Genetic Structure

Beetles and fungi exhibited similarly weak genetic structure (*Fst* = 0.036 and 0.039, respectively) across the entire study area (Table 2). In the northern region, beetles had very little genetic structure (*Fst* = 0.01) whilethe fungus resembled the entire study area (*Fst* = 0.034). Both species were very weakly structuredin the southern region (*Fst*<0.01). The 95% confidence intervals around these *Fst* estimates did not include zero for the total and northern regions (Table 2). However, in the southern region, the confidence intervals around *Fst* estimates for both species did include zero and indicate non-significant genetic structure.

**Table 2:** Summary of global *Fst* for *D. ponderasae* and *G. clavivera* in different sample regions.“North” and “South” refer to regions in Figure 2.

		Fungus		Beetle	
	*n*	Fst	95% CI	Fst	95% CI
**All**	**15**	0.036	0.002–0.090	0.039	0.026–0.053
**North**	**8**	0.034	0.003–0.084	0.010	0.006–0.014
**South**	**7**	0.009	−0.028–0.051	0.002	0.000–0.003

n refers to the number of landscapes used for calculation. Confidence intervals that include zero indicate non-significant structure.

### Isolation by distance

**Figure 3:**
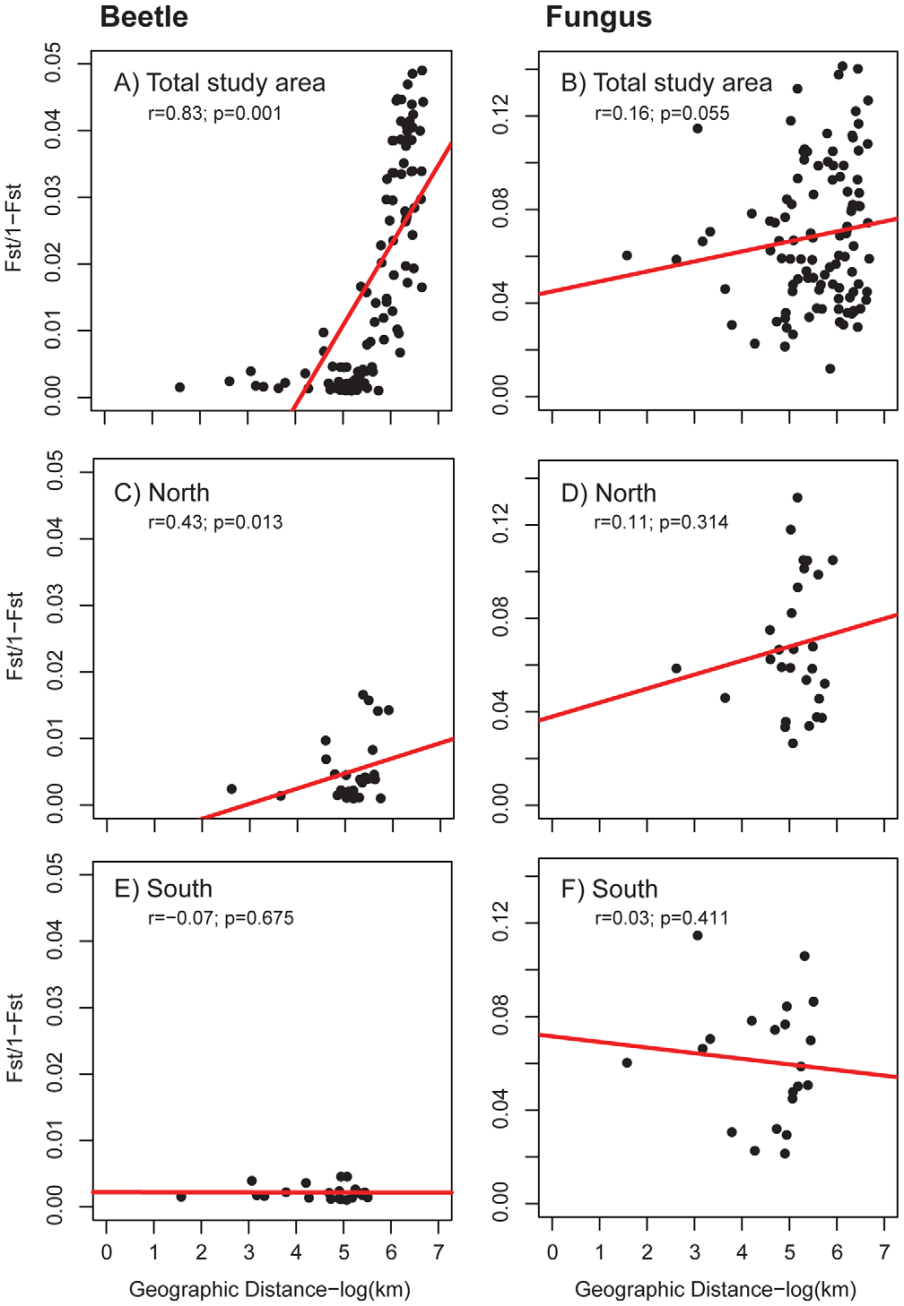
Isolation by distance plots. Genetic distance is plotted as a function of geographic distance for beetles (left columns) and fungus (right column) for the entire study area (A & B), the northern region (C & D), and the southern region (E & F). Note the different scales for the beetle and fungal plots. Red lines show the best fit line to the data and are included for illustration only. *r* values represent correlation between geographic and genetic distances matrices assessed using Mantel tests.

We identified significant IBD for the entire study area for both beetles and fungi (Figure 3a, 3b) based on Mantel tests of correlation between matrices of geographic and genetic (Nei's *Fst*) distance. However, much of the structure identified was related to comparisons between regions. When examining each region separately, we found IBD for the beetles in the north, but not for the fungus (Figure 3c, 3d). IBD was not detected for either species in the south (Figure 3e, 3f).

### Analysis of Molecular Variance

The majority of genetic variation for the beetle was found within individuals (91.2%, *p*<0.001; AMOVA; Table 3), whereas the majority of genetic variation for the fungus was foundamong individuals within landscapes (92.5%, *p*<0.001; AMOVA; Table 3). For both taxa, variation between regions was also significant and accounted for 6.04% and 8.6% of genetic variation in beetles and fungus, respectively (AMOVA; Table 3).

**Table 3:** AMOVA summaries that describe the proportion of genetic variance in the fungus and the beetles at different hierarchical levels.

Fungus					
Source	df	SS	Variance	Percent	*p*-value
Between regions	1	36.159	0.210	8.628	<0.001
Among landscapeswithin regions	14	55.177	−0.031	−1.271	0.903
Among individuals within landscapes	139	626.806	2.247	92.469	<0.001
**TOTAL**	309	718.142	2.430		

Beetle					
Source	df	SS	Variance	Percent	*p*-value
Between regions	1	651.615	0.245	6.054	<0.001
Among landscapeswithin regions	15	145.203	0.020	0.497	<0.001
Among individuals within landscapes	2600	10063.182	0.089	2.207	<0.001
Within individuals	2617	9661.500	3.692	91.241	<0.001
**TOTAL**	5233	20521.500	4.046		

Regions refer to northern and southern (Figure 2; Table S1). Variation within individuals is not reported for the fungus because it is haploid.

Fungi sampled from larvae within galleries were less genetically structured (*Fst* = 0.039) than fungi from wood tissue (*Fst* = 0.086). However, the proportion of variance explained by the fungal source (wood vs. fungus) was not significant (AMOVA; σ^2^ = 0.0001; *p* = 0.391). Therefore, fungal data from both sources were pooled for further analysis.

Year of sampling did not explain any significant variation in the fungus (AMOVA; σ^2^ = −0.0004; *p* = 0.74) or in the beetle (AMOVA; σ^2^ = −0.0002; *p* = 0.93) in the four sites that were sampled in both periods. Here, the negative variance (σ^2^) simply indicates that the predictor performs more poorly than random values. Therefore, differences among sites are not due to differences between sample periods for both fungi and beetles (Table S1).

### Spatial genetic structure

**Figure 4:**
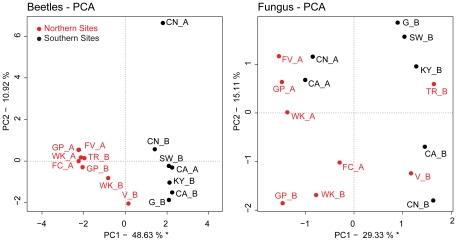
Principal Components Analysis (PCA) of raw allele frequency data. Plots of site scores for the first two principal components are shown for (a) beetles, and (b) fungi. Percentage values associated with each axis represent the respective proportion of overall variance in allele frequencies captured.

The first beetle principal component indicated a very strong north-south gradient in beetle genetic structure (Figure 4a) and accounted for 48.6% of the variation in the allele frequency data. The second axis captured 10.9% of the variation. This strong pattern of association between location and genetic structure was not present in the fungus (Figure 4b). Here, the first axis captured only 13.23% of the variation and the second axis captured 10.27% (Figure 4b). Visual inspection of factor loadings did not suggest that any single allele was disproportionately influencing the ordinations. Principal coordinates analysis using *Fst* (not shown) were highly correlated with the ordination solutions from the PCA. Fungal variation was more similarly described by the two methods than the beetles except in the southern region. Correlations between the PCA and PCoAfor the beetles were equal to 0.77 (*p* = 0.001), 0.898 (*p* = 0.001), and 0.818 (*p* = 0.027) in the total, northern, and southern regions, respectively. Correlations for the fungus were equal to 0.90 (*p* = 0.001), 0.93 (*p* = 0.001), and 0.79 (*p* = 0.03) also for the total, northern, and southern regions, respectively.

Latitudeaccounted for a significant proportion of the beetle allele frequency variation acrossthe entire study area (RDA; *R^2^_adj_* = 0.389, *F* = 9.89, *p* = 0.005), but not for fungi (*R^2^_adj_* = 0.032, *F* = 1.466, *p* = 0.130). This same pattern was found in both sub-regions. In the north, beetle genetic variation covaried with latitude (*R^2^_adj_* = 0.138, *F* = 2.12, *p* = 0.025) but fungalvariation did not (*R^2^_adj_* = 0.013, *F* = 1.09, *p* = 0.34). We also found this pattern in the south for beetles (*R^2^_adj_* = 0.115, *F* = 1.78, *p* = 0.01) and fungi (*R^2^_adj_* = 0.058, *F* = 1.36, *p* = 0.107).

### Concordance in beetle and fungal genetic structure

Procrustes rotation on the PCA ordination solutions, in which the effect of spatial locationwas present, showed correlation of allele frequencies between taxa, bothin the total study area and in the southern region (Table 4). Afterremoving the effect of Euclidean connectivity among locations, only the relationship at the level of the total study area was significant. Genetic variation of beetles and fungus was also correlated based on PCoA ordinations of *Fst*-values at the level of the total study area. Similar to the PCA comparisons, that correlation was not significant once the effects of Euclidean distance were removed. However, unlike the comparisons based on PCA, no correlation was identified between beetle and fungal PCoAs in the southern region (Table 4).

**Table 4:** Results from Procrustes rotation tests to determine the strength and significance of correlation between spatial patterns in beetle and fungal allele frequencies.

	PCA				PCoA			
Data	*T*	*p*	*t′*	*p′*	*t*	*p*	*t′*	*p′*
**All**	0.498	**0.040**	0.381	0.263	0.487	**0.038**	0.344	0.354
**North**	0.450	0.498	0.487	0.431	0.393	0.670	0.442	0.545
**South**	0.811	**0.024**	0.737	**0.046**	0.657	0.140	0.607	0.202

t represents correlation between matrices and p represents the significance of that correlation. t′ and p′ represent the same correlation between matrices in which the effects of Euclidean distance were controlled for. PCA refers to comparisons between ordination solutions using Euclidean distances and PCoA refers to ordination solutions from PCoA based on Fst values. Significant correlations are in bold.

### Landscape Genetics

The different ordination methods also identified similar spatial predictors of genetic variation for both species (Table 5). In the entire study area, a model of connectivity based on Euclidean distance (IBD) best explained spatial genetic variation regardless of genetic distance employed. However, the amount of variance explained was generally low (*R^2^adj*<0.14; Table 5). In the northern region, the best beetle dbRDA model included pine volume (*R^2^adj* = 0.35), whereas the best beetle RDA model included both pine volume and climate (*R^2^adj* = 0.32). The unique contributions (*R^2^adj*) of climate and pine volume were 12.8% and 9.6%, respectively, with 9.1% of the variation explained shared between the two predictors. For both approaches, nothing predicted fungal genetic variation. Models in the southern region differed between methods. Using dbRDA, nothing accounted for beetle genetic variation, whereas using RDA, fungal genetic variation (PC1) was a significant predictor (*R^2^adj* = 0.11; Table 5). Conversely, RDA did not identify any significant predictors for the fungus, but the best dbRDA model included both Euclidean distance and beetle genetic structure (PCo2; *R^2^adj* = 0.33; Table 5), which had unique contributions (*R^2^adj*) of 9.2% and 9.3%, respectively, with 14.6% of the variation explained shared.

**Table 5:** Summary of model selection on constrained ordination models (RDA and dbRDA) that describe the influence of spatial heterogeneity on beetle and fungal genetic variation.

RDA	Region	Species	Model	*F*	*p-value*	*R^2^*	*R^2^_adj_*
	**ALL**	Beetle	∼Euclidean Distance	2.804	0.034	0.177	0.114
	*n* = 15	Fungus	∼Euclidean Distance	2.680	0.007	0.171	0.107
	**NORTH**	Beetle	∼CSI+Pine Volume	2.614	0.005	0.511	0.316
	*n* = 8	Fungus	-	-	-	-	-
	**SOUTH**	Beetle	∼Fungi PC2	1.743	0.025	0.258	0.110
	*n* = 7	Fungus	-	-	-	-	-

dbRDA	Region	Species	Model	*F*	*p-value*	*R^2^*	*R^2^_adj_*
	**ALL**	Beetle	∼Euclidean Distance	1.953	0.028	0.131	0.064
	*n* = 15	Fungus	∼Euclidean Distance	3.175	0.005	0.196	0.134
	**NORTH**	Beetle	∼Pine Volume	4.737	0.015	0.441	0.348
	*n* = 8	Fungus	-	-	-	-	-
	**SOUTH**	Beetle	-	-	-	-	-
	*n* = 7	Fungus	Euclidean Distance+Beetle PCo2	2.463	0.015	0.552	0.328

For each species, forward selection was applied to identify which variables best described variation in allele frequencies using an inclusion threshold of α = 0.05. Significant correlations are in bold. Dashes indicate that no variables were selected.

## Discussion

### Genetic Structure

Given the symbiotic relationship between the mountain pine beetle and *G. clavigera* we expected the genetic structure of these two species to be similar. This was the case when we examined the entire study area (*Fst*∼0.04) but species differed within regions. The fungus retained its structure in the north, while the beetle did not. Both species demonstrated very weak genetic structure in the south (*Fst*<0.01). Similarly, the correlations in genetic structure between species were not consistent between regions or between methods (Table 4, Table 5). Correlations at the level of the entire study area confirmed our expectations, as did the finding that this correlation was largely due to shared spatial dependence. In the south, significant correlation between species persisted even after controlling for spatial structure when using Euclidean distance between allele frequencies; however, no correlation was identified using *Fst* and it remains puzzling why we did not find any correlation in the north where both species have recently expanded. We found significant IBD when examining the entire study area but also found that this pattern was largely due to differences between regions in both species. That the two species differed among regions suggests that different processes may be responsible for governing gene flow in these symbiotically interacting species.

Beetle and fungus allele frequencies respond differently to environmental heterogeneity (Table 4 and Table 5). Most importantly, beetles demonstrated a strong geographical trend between regions whereas the fungus did not (Figure 3, Figure 4). Using PCA, we clearly demonstrated the divide between regions in the beetle (Figure 4a); almost 40% of the beetle variation can be described by latitude. The division between north and south regions was also demonstrated using AMOVA (Table 3) and the decrease in beetle heterozygosity from south to north (Table S1). Bootstrapped rarefaction of the beetle genetic data to a set of samples similar in size to the fungus still revealed a strong response to latitude (*datanot shown*). Previous studies of the concordance of genetic structure between symbionts have suggested that different scales of dispersal between species may account for the incongruence in spatial genetic variation [16]. In this case, this explanation seems unlikely because it is generally thought that *G. clavigera*does not disperse independently of the beetle [10]. Nonetheless, because the cryptic sexual stage of this fungus is rarely observed, there may yet be more to discover regarding the dispersal of sexual spores [23], [47].

### Landscape Genetics

When examining the study area as a whole, Euclidean connectivity was found to best predict genetic variation in both species, likely due to the strong differences between regions. Similarly, landscape genetic models differed between regions for both methods used and within regions, the influence of environmental heterogeneity on genetic variation differed between species. In the north, pine volume significantly predictedbeetle genetic connectivity using both methods. Using RDA, which does not make any evolutionary assumptions, climate was also identified as a significant predictor. In the south we found some evidence for significant reciprocal genetic influences, although these influences were not symmetrical between methods: the fungus helped explain the beetle using RDA, but beetles helped explain the fungus using dbRDA.

The differences between regions have most likely arisen because each region has had a different period of association with the mountain pine beetle and different processes determine beetle and fungal movement and hence spatial genetic variation. The northern region was recently colonized and is at the front of the current outbreak [24], [44]. In contrast, the southern region has had a long history with the beetle under both endemic and epidemic conditions [24], [48]. Presumably, the longer association with the beetle in the south also means a longer association with the fungus. These differences mean that the northern region is likely farther from mutation-drift equilibrium than the south, and suggests that a non-evolutionary distance metric based on allele frequencies (e.g., PCA), is more appropriate for analysis. In addition to representing different spatial contexts, the regions may represent different temporal stages of the outbreak system that proceeds from initial colonization to the development of correlated spatial genetic structure over time. Thus, in the south, *Fst* may be more appropriate as a distance measure as its assumptions are more likely to be met. Differences between the two methods in the south may reflect these differences in population genetic structure, history of association, or temporal stage of the outbreak. Interestingly, *Fst* and Euclidean distance perform similarly in the area of recent expansion.

With specific regard to the fungus, the different structure between regions can also explained by spatial variation in MPB-associated fungal community and the relative frequency of *G. clavigera* in northern Alberta. Recent work on the MBP-associated fungal community found that *G. clavigera* is typically found in the south, whereas *L. longiclavatum*, is more typically found in the north of Alberta [49]. In light of this work, it is plausible that the genetic differences between regions and between species are the result of a recent range expansion as wellas a relatively small population of *G. clavigera* in the north. It will be valuable to contrast these results with those from other fungal species.

In the north, pine volume affectedbeetle spatial genetic variation (Table 5) according to both RDA and dbRDA models. Host connectivity has been previously shown to play an important role in the rate of spread of outbreaks [24], [50]. Pine volume is important because MPB preferentially attack large trees and greater host connectivity means that dispersing beetles are more likely to find high volume regions to produce brood and facilitate further spread [24], [51]. These results further indicate that management of host connectivity is important for managing pine beetle outbreaks, and specifically, this connectivity may need to be managed at large spatial scales. However, it remains to be investigated whether host availability affects genetic connectivity also at fine spatial scales and highlights the importance of considering scale in landscape genetic studies [52].

Climate also explained a significant proportion of variance in beetle genetic variation in the northern region, but only in the RDA model (Table 5). In this context, climate represents the connectivity of all sites to the other sites based on a spatial surface of climatic suitability [38]. The current expansion and large scale outbreak in western Canada has been previously attributed to changes in climatic conditions [2], [53]. Here, using neutral genetic data, we were able to demonstrate that at the leading edge of the outbreak, and assuming non-equilibrium conditions, sites separated by less resistance in terms of climate tend to have more similar beetle genetic structure, but the fungus did not exhibit the same relationship.

### Genetic Interactions

Genetic correlations were identified in the southern region using both ordination approaches. Using RDA, asignificant proportion of variation in beetle allele frequencies (∼11%) was explained by the first fungus PCA axis (PC1). There was not an equivalent effect of beetles on the fungus. The opposite effect was found using dbRDA and the beetles, in combination with Euclidean distance, significantly described the fungus. Thesefindings represent an important aspect of the symbiotic relationship that we identified using the integrated landscape genetics approach. Most likely, these effects represent either an unmeasured environmental variable to which both species respond or demographic interactions between species and highlights the utility of using additional biotic information in landscape genetic studies. Previous work has demonstrated the importance of host plant genotypes on the dynamics of associated insect herbivores [54] and plant pathogens [55]. Although we did not examine adaptive genetic variation, our findings suggest that similar genetic interactions may play a role in paired symbiont dynamics and gene flow. That we found these relationships exclusively in the south further supports our assertion that the processes governing gene flow are different or at different temporal stages between the two regions. It will be worthwhile to examine the northern region in the future to assess whether concordance develops over time to a level similar to that of the south.

### Pathways

All pathways (Figure 1) were supportedin some capacity but varied in terms of their relative strengthsand the regions in which they were supported (Table 5). Most important was the relationship between Euclidean distance and genetic variation in both species in the entire study area and that the supported pathways differed between regions. Pathways 3 and 4 (genetic correlations) were only present in the south and were not equivalent between methods. The relatively low variance (*R^2^adj*) explained by the environment suggests that landscape resistancemay not be the most suitable model for examining the landscape genetics of a highly mobile species such as the mountain pine beetle, at least at the spatial and temporal scale we examined. Indeed, much of the genetic variation resides within individuals and within sample sites (AMOVA; Table 3). Furthermore, uncertain long distance dispersal events may obviate the influence of landscape heterogeneity on genetic variability during a large outbreak. Continued immigration from northern BC may act to effectively “reshuffle the genetic deck” and confound our efforts to understand gene flow at local scales. Finally, differences in generation time and sexual systems between taxa could mean that the genetic markers used in this study capture different ecological and evolutionary processes.

### Methodological considerations

We used constrained ordination to examine relationships among allele frequencies (RDA) or genetic distance (dbRDA) and environmental connectivity, and dimensionally reduced symbiont genetic variation. Recently, Balkenhol et al. [56] reviewed statistical methods of landscape genetic analysis and identified constrained ordination as one of the more powerful methods of analysis. Analyses based on correlations among distance matrices (e.g., Mantel tests) have been shown to be less powerful for linear modelling than ordination-based methods [32], [42]. Furthermore, our objective was to partition genetic variance and to assess the relative contributions of different environmental and biological factors. Constrained ordinationsupportedcalculation of unbiased adjusted R-squared values for each model and each component within models [57]. Finally, these methods allowed us to investigate the genetic correlations between species in a robust and integrated way using principal components and principal coordinates. This framework is general and can be used to analyse many different types of genetic data and can include any measure of dissimilarity among sample locations. Some challenges remain to decide how to convert different types of genetic data such as SNP or genomic sequence data into a format suitable for variance decomposition using this approach.

We assessed concordance in genetic patterns and gene flow between the mountain pine beetle and *G. clavigera* but did not address whether the two species are adaptively responding to each other or their environment. This study represents one of the first to use a biological predictor, specifically genetic information, to describe genetic variation of an interacting species in a landscape genetics framework. Future work using this integrated framework will assess adaptive genomic interactions among taxa in the mountain pine beetle system as well as concordance in evolutionary responses to environmental heterogeneity. Opportunities also exist to further investigate the relative strengths of different measures of genetic distance (e.g., Euclidean distance vs. *Fst*) for describing landscape genetic relationship in different spatial contexts. Ongoing research to identify SNP markers and to characterize functional genes in the beetle, the fungus, and host pine trees will be used to investigate these relevant evolutionary questions in a spatially explicit and integrated context.

### Conclusions

We presented a novel integrated landscape genetics framework to investigate reciprocal genetic correlationsbetween species while also testing for the influence of spatial heterogeneity. Contrary to expectation, we found differences in genetic structure between the mountain pine beetle and *G. clavigera.* We also identified important differences in the genetic congruence between species within the historical southern range of the MPB that depend on the assumptions underlying different measures of genetic distance. Finally, we identified genetically-based support for the role of host connectivity (pine volume) and climate in the spread of the outbreak in the north. The different patterns of genetic structure between regions likely reflect the different processes that determine endemic vs. epidemic population structure and the recent northward population expansion. The next challenge will be to examine the three-way genetic correlationsamong the beetle, its fungal symbionts, and its host pine trees.

## Supporting Information

Table S1 **Summary of all sample sites arranged from north to south.** Site names include the location followed by a letter indicating the unique site within the sample landscape. Single letters after the underscore indicate year sampled: “_A” represents a sample from Feb.–May 2007; “_B” represents a sample from Sept.–April 2008. Elevation is indicated in metres above sea level.
(DOCX)
