# Correction: Spatial Genetic Structure of a Symbiotic Beetle-Fungal System: Toward Multi-Taxa Integrated Landscape Genetics

**DOI:** 10.1371/annotation/33c0f48a-2519-4621-b7b8-a9bf84502ce5

**Published:** 2013-10-25

**Authors:** Patrick M. A. James, Dave W. Coltman, Brent W. Murray, Richard C. Hamelin, Felix A. H. Sperling

PLOS ONE introduced several formatting errors during the conversion of this article for the production process resulting in several words not being properly spaced apart. You can find the original manuscript here: http://www.plosone.org/corrections/pone.0025359.001.cn.docx

