# Supplementary material for: Spatial Genetic Structure of a Symbiotic Beetle-Fungal System: Toward Multi-Taxa Integrated Landscape Genetics
Source: PLoS One. 2011 Oct 4;6(10):e25359. doi: 10.1371/journal.pone.0025359 (PMC3186778; doi:10.1371/journal.pone.0025359)
Supplement: Table S1 — Summary of all sample sites arranged from north to south. Site names include the location followed by a letter indicating the unique site within the sample landscape. Single letters after the underscore indicate year sampled: “_A” represents a sample from Feb.–May 2007; “_B” represents a sample from Sept.–April 2008. Elevation is indicated in metres above sea level. (DOCX) [file pone.0025359.s001.docx]

**Table S1**

| **Landscape** | **Label** | **Region** | **Lat.** | **Long.** | **Elev.** | **Beetle N^1^** | **Beetle *H*_E_^2^** | **Fungus N^1^** | **Fungus *H*_E_^2^** |
| --- | --- | --- | --- | --- | --- | --- | --- | --- | --- |
| **Fairview** | FV_A | North | 56.23 | -119.26 | 652 | 78 | 0.46 | 11 | 0.54 |
| **Tumbler Ridge** | TR_B | North | 55.24 | -121.46 | 1114 | 308 | 0.45 | 13 | 0.56 |
| **Grand Prairie** | GP_A | North | 55.05 | -118.70 | 622 | 157 | 0.45 | 14 | 0.58 |
| **Grand Prairie** | GP_B | North | 54.84 | -119.16 | 817 | 277 | 0.46 | 6 | 0.66 |
| **Fox Creek** | FC_A | North | 54.66 | -116.65 | 830 | 41 | 0.46 | 11 | 0.61 |
| **Willmore - Kakwa** | WK_A | North | 53.75 | -119.75 | 1821 | 69 | 0.47 | 9 | 0.58 |
| **Willmore - Kakwa** | WK_B | North | 53.79 | -119.55 | 1598 | 174 | 0.50 | 8 | 0.55 |
| **Valemount** | V_B | North | 52.90 | -119.35 | 825 | 197 | 0.56 | 8 | 0.58 |
| **Golden** | G_B | South | 51.34 | -116.82 | 2107 | 274 | 0.60 | 7 | 0.56 |
| **Kootenay-Yoho** | KY_B | South | 51.12 | -116.29 | 2177 | 153 | 0.62 | 7 | 0.57 |
| **Canmore** | CA_A | South | 50.99 | -115.36 | 1824 | 97 | 0.61 | 10 | 0.59 |
| **Canmore** | CA_B | South | 51.00 | -115.29 | 2240 | 375 | 0.61 | 29 | 0.52 |
| **Crowsnest Pass** | CN_A | South | 49.64 | -114.59 | 1370 | 27 | 0.61 | 4 | 0.63 |
| **Crowsnest Pass** | CN_B | South | 49.83 | -114.55 | 1748 | 72 | 0.62 | 5 | 0.66 |
| **Sparwood** | SW_B | South | 49.81 | -114.88 | 1183 | 217 | 0.61 | 7 | 0.55 |

^1^ – Number of individual beetle or fungal single spore isolates sampled

^2^ – Nei’s unbiased expected heterozygosity
